# CircLRP6 contributes to prostate cancer growth and metastasis by binding to miR-330-5p to up-regulate NRBP1

**DOI:** 10.1186/s12957-021-02287-2

**Published:** 2021-06-22

**Authors:** Linghui Qin, Xiaosong Sun, Fei Zhou, Cheng Liu

**Affiliations:** Department of Urology, Xiangyang Central Hospital, Affiliated Hospital of Hubei University of Arts and Science, No. 136, Jingzhou Street, Xiangyang, 441021 Hubei China

**Keywords:** circLRP6, MiR-330-5p, NRBP1, Prostate cancer, Metastasis, Growth

## Abstract

**Background:**

Circular RNA low-density lipoprotein receptor-related protein 6 (circLRP6) is considered as an oncogene in many types of cancers. However, the function and mechanisms of circLRP6 in prostate cancer (PCa) tumorigenesis remain largely undefined.

**Methods:**

Quantitative real-time polymerase chain reaction (qRT-PCR) and western blot assays were conducted to assess the expression of circLRP6, microRNA (miR)-330-5p, and nuclear receptor binding protein 1 (NRBP1). Cell counting kit-8 (CCK-8), colony formation, 5-ethynyl-2’-deoxyuridine (EDU) incorporation, flow cytometry, transwell, wound healing, and western blot assays were performed to detect cell proliferation, apoptosis, and metastasis in vitro. Subcutaneous tumor growth was observed in nude mice to investigate the role of circLRP6 in vivo. The targeting relationship between miR-330-5p and NRBP1 or circLRP6 was verified using dual-luciferase reporter, pull-down, and RNA immunoprecipitation (RIP) assays. Immunohistochemistry was employed to test relative protein expression.

**Results:**

CircLRP6 was highly expressed in PCa tissues and cells, knockdown of circLRP6 impaired PCa cell growth and metastasis in vitro by affecting cell proliferation, apoptosis, invasion, migration, and epithelial-mesenchymal transition (EMT). Mechanistic studies showed that circLRP6 could competitively bind with miR-330-5p to prevent the degradation of its target gene NRBP1. Rescue assay suggested that miR-330-5p inhibition reversed the inhibitory effects of circLRP6 knockdown on PCa cell growth and metastasis. Moreover, overexpression of miR-330-5p suppressed PCa progression via NRBP1. Notably, tumor formation assay indicated that circLRP6 silencing impeded tumor growth and EMT in vivo.

**Conclusion:**

Our findings demonstrated that circLRP6 promoted PCa tumorigenesis and metastasis through miR-330-5p/NRBP1 axis, suggesting a potential therapeutic target for PCa.

**Supplementary Information:**

The online version contains supplementary material available at 10.1186/s12957-021-02287-2.

## Background

Prostate cancer (PCa) is the most common malignancy in men through the world, with an estimated 1.6 million new cases and 366,000 deaths annually [1, 2]. Despite the high long-term survival in localized PCa and the effectivity of androgen deprivation therapy in the control of metastatic PCa, the majority of patients still develop castration-resistant PCa (CRPCa) [3–5]. Thus, further investigations on the pathogenesis and identifications on novel bio-labelling therapy in PCa are of great significance.

Currently, accumulating evidence has suggested that noncoding RNAs, including circular RNAs (circRNAs), microRNAs (miRNAs/miRs), and long noncoding RNAs (lncRNAs), impact a variety of biological processes, such as cell proliferation, apoptosis, and metastasis [6]. Unlike linear RNAs, circRNAs possess covalently closed loop structures that lack the 3′ and 5′ ends, and are highly stable relative to their linear counterparts in vivo due to the resistance to the degradation by RNA exonuclease [7, 8]. Besides that, most of circRNAs are highly conserved across species and exhibit development- or tissue-specific expression pattern [9]. Importantly, recent reports have proved the involvement of circRNAs in cancer progression by acting as carcinogens or tumor suppressors [10, 11]. Thus, circRNAs may be promising therapeutic targets for cancers. In PCa, some circRNAs, such as circFoxo3 [12], circUCK2 [13], and circ_0044516 [14], were also found to be aberrantly expressed and functioned as a critical type of endogenous RNAs to regulate cancer cell survival, invasion, migration and therapeutic resistance, thus affecting PCa tumorigenesis. CircRNAs low-density lipoprotein receptor-related protein 6 (circLRP6) (ID: hsa_circ_0000378) is derived from exons 2 of the LRP6 gene. A previous study suggested that highly expressed circLRP6 was related to the worse overall survival in osteosarcoma, and circLRP6 promoted osteosarcoma tumorigenesis by downregulating KLF2 and APC expression level [15]. Xue et al. revealed that circLRP6 enhanced arsenite-evoked malignant transformation in human keratinocyte cells by inducing cell epithelial-mesenchymal transition (EMT) via miR-455/ZEB1 axis [16]. Besides, circLRP6 was also found to upregulate Myc expression level by targeting miR-182 to promote cell proliferation and invasion in esophageal squamous cell cancer [17]. Therefore, circLRP6 may function as an oncogene in cancer progression.

In this study, we speculated that circLRP6 might also serve as a carcinogen in PCa progression. Here, the action of circLRP6 in PCa tumorigenesis in vitro and in vivo was investigated. Besides that, how circLRP6 affected the tumorigenesis of PCa was further explored.

## Materials and methods

### Clinical specimens

Seventy-one tissue samples, including 19 normal prostate tissues and 52 PCa tissues, were collected from PCa patients admitted to Xiangyang Central Hospital, and then stored at – 80 °C until further used. All specimen diagnoses were confirmed by pathological examinations after surgery. The study equipped with written informed consent from each enrolled individual.

### In situ hybridization (ISH)

Paraffin-embedded tissues from PCa patients were collected. CircLRP6-positive expression was examined using a specific digoxigenin (DIG)-labeled circLRP6 probe designed by Geneseed (Guangzhou, China). After being dewaxed in xylene and rehydrated through gradient alcohol, the tissue microarray (TMA) was digested using proteinase K and hybridized with the specific circLRP6 probe overnight at 4 °C, followed by incubation with anti-DIG-AP (Roche, Basel, Switzerland) overnight 4 °C. After staining with nitro blue tetrazolium (NBT)/5-bromo-4-chloro-3-indolyl phosphate (BCIP) (Roche), the staining results were observed.

### Cell culture

Human RWPE-1 cells, human PCa cell lines (PC3 and DU145), and 293 T cell lines were purchased from Cedarlane (Burlington, NC, USA). All cells were cultivated in RPMI-1640 medium (Sigma, St. Louis, MO, USA) plus penicillin/streptomycin (100 U/mL, Gibco, Rockville, MD, USA) and 10% fetal bovine serum (FBS, Gibco) at 37 °C with 5% CO_2_.

### Quantitative real-time polymerase chain reaction (qRT-PCR)

The isolation of total RNA was conducted using the TRIzol (Life Technologies, Waltham, MA, USA). Total RNA (2 μg) was incubated for 30 min at 37 °C with or without 3 U/μg of RNase R (Epicentre Technologies, Madison, WI, USA) to verify the circular characteristics of circRNA. Reverse transcription was performed using the PrimeScript RT Reagent Kit (Takara, Dalian, China) under recommended condition. Then SYBR® Select Master Mix (Takara) was employed to perform qRT-PCR. U6 or glyceraldehyde-3-phosphate dehydrogenase (GAPDH) was used as the normalization control and the expression levels of molecules were examined using the 2^−ΔΔCt^ method. The primer sequences for qRT-PCR were listed:

circLRP6: F 5′-GAGTTGGATCAACCCAGAGC-3’, R 5′-TCCTCCAAGCCTCCAACTAC-3′;

Nuclear Receptor Binding Protein 1 (NRBP1): F 5′-GAGGTGAATCAACGGAATGTACC-3′, R 5′-CTTGTAGTTCTTGCGTTCAGAGA-3′;

LRP6: F 5′-TTTATGCAAACAGACGGGACTT-3’, R 5′-GCCTCCAACTACAATCGTAGC-3′;

GAPDH: F 5′-CCCACATGGCCTCCAAGGAGTA-3′, R 5′-GTGTACATGGCAACTGTGAGGAGG-3′;

miR-330-5p: F 5′-GGGACACAGGGCCAGAGAC-3′, R 5′-GGGACACAGGGCCAGAGAC-3′;

U6: F 5′-CTCGCTTCGGCAGCACA-3′, R 5′-AACGCTTCACGAATTTGCGT-3′.

### Cell transfection

The siRNA duplexes targeting circLRP6 (si-circLRP6#1, si-circLRP6#2 and si-circLRP6#3) and scrambled siRNA (si-NC), pcDNA3.1 NRBP1 overexpressing plasmid (pcDNA-NRBP1) and negative control (pcDNA), miR-330-5p mimic, miR-330-5p inhibitor, mimic-control (mimic NC), and inhibitor-control (inhibitor NC) were obtained from GenePharma (Shanghai, China). The PC3 and DU145 cells grown to 60–70% confluence were transfected with miRNA mimics, miRNA inhibitors, plasmids siRNAs, or respective controls using Lipofectamine 2000 (Invitrogen, San Diego, CA, USA).

### Cell proliferation assays

Cell proliferation was determined by cell counting Kit-8 (CCK-8), colony formation, and 5-Ethynyl-2′-Deoxyuridine (EDU) incorporation assays. For CCK-8 assay, transfected PC3 and DU145 cells were placed into 96-well plates with 3 × 10^3^ per well. At 24, 48, or 72 h incubation, each well was added with 10 μL of CCK-8 reagent (Beyotime, Shanghai, China) and incubated for another 1 h. The absorbance in each well was measured using a microplate reader at 450 nm (Bio-Rad, Hercules, CA, USA). For colony formation assay, PC3 and DU145 following assigned transfection were plated at 6-well plates (500 cells/well). After culture for 14 days, cell colonies were fixed with ethanol (Beyotime), and the number of colonies was imaged and counted after crystal violet staining. For the EDU incorporation assay, an EDU incorporation assay kit (RiboBio, Guangzhou, China) was used. Transfected PC3 and DU145 cells were incubated with 50 mM EDU for 2 h. Then, cells were fixed with 4% paraformaldehyde (Beyotime) and stained with Apollo Dye Solution for proliferating cells. Subsequently, the nuclei were stained with DAPI. The images were obtained employing a fluorescence microscope, and stained cells were analyzed using ImageJ software (National Institutes of Health, Sacaton, AZ, USA).

### Cell cycle and apoptosis analysis

Transfected PC3 and DU145 cells were trypsinized and rinsed with pre-cold phosphate buffer solution (PBS). Cell supernatant was discarded after centrifugation, and then cells were fixed with 70% cold ethanol for 1 h. After washing with PBS, fixed cells were stained with propidium iodide (PI) (BD Biosciences, Franklin Lakes, NJ, USA), and the FACScan flow cytometer (BD Biosciences) was applied to determine cell cycle distribution.

For cell apoptosis analysis, transfected PC3 and DU145 cells were resuspended in 500 μL 1× Annexin binding buffer to obtain a concentration of 5 × 10^5^/mL, followed by staining with Annexin V-fluorescein isothiocyanate (FITC) (BD Biosciences) (5 μL) and propidium iodide (PI) (10 μL). Cell apoptosis was detected using the FACScan flow cytometry (BD Biosciences).

### Cell invasion and migration analysis

Cell invasion was analyzed by transwell chambers (pore size 8 μm; Costar, Cambridge, MA, USA) with matrigel-coated filters (BD Biosciences). Transfected PC3 and DU145 cells (2 × 10^5^ cells) were plated in the top chamber. The lower chamber contained 600 μL medium with 10% FBS was used as a chemoattractant. The cells were incubated at 37 °C with 5% CO_2_ for 24 h. The number of invaded cells in five random fields of the lower surface was counted after crystal violet staining.

Cell migration was analyzed using wound healing assay. After transfection, PC3 and DU145 cells were incubated in a 6-well plate containing RPMI-1640 plus 10% FBS. When cells grown to a fully confluent monolayer, cells were scratched using a 200-μL pipette tip and then grown in the serum-free medium. At 0 and 24 h, the width of wounds was detected and captured.

### Western blot

Total protein was extracted with RIPA buffer and the protein concentration was quantified using a BCA protein assay kit (Com Win Biotech, Qingdao, China). A total of 50 μg protein was separated by 10% SDS-polyacrylamide gel electrophoresis, and then transferred onto the Clear Blot membrane-p (ATTO, Tokyo, Japan). The blots were incubated with anti-E-Cadherin (ab1416), anti-Vimentin (ab8978), anti-GAPDH (ab181602) (all 1:1000; Abcam, Shanghai, China), and anti-NRBP1 (Cat# H00029959-M01, 1:1000, Abnova, Taipei, Taiwan) overnight at 4 °C and then incubated with HRP-labeled specific secondary antibody for 1 h at room temperature. Protein blots were visualized with an enhanced chemoluminescence kit (KeyGen) and scanned using ImageJ software based on the mixture of both intensity and band thickness.

### Dual-luciferase reporter assay

The bindings between miR-330-5p and NRBP1 or circLRP6 was predicted by starBase3.0 (http://starbase.sysu.edu.cn/) or Circinteractome database (https://circinteractome.nia.nih.gov/), respectively [18]. Wild-type plasmids circLRP6 wt and NRBP1 wt, and mutant-type plasmids circLRP6 mut and NRBP1 mut were inserted into the pGL3 promoter vector (GenePharma, Shanghai, China). 293T cells were seeded into 24-well plates and co-transfected with 100 ng of reporter plasmids, 20 ng of pRL-TK Renilla Luciferase vector and 50 nM miRNAs mimics/negative control using Lipofectamine 2000 (Invitrogen). A dual-luciferase reporter assay kit (Promega, Madison, WI, USA) was employed to analyze the luciferase activity.

### RNA immunoprecipitation (RIP) assay

PC3 and DU145 cells were firstly lysed in RIP lysis buffer (Millipore, Billerica, MA, USA) and then incubated with RNA magnetic beads (Millipore) conjugated with Anti-Ago2 (ab32381, Abcam) or anti-IgG. At last, the immunoprecipitated RNAs were eluted, purified and subjected to qRT-PCR.

### Pull-down assay

Biotinylated miR-330-5p (Bio-miR-330-5p) and its negative control (Bio-NC) were generated by Sangon (Shanghai). PC3 and DU145 cells were lysed and the lysate was incubated with streptavidin-coated magnetic beads containing Bio-miR-330-5p or Bio-NC. Following being eluted, the levels of circLRP6 and NRBP1 were detected using qRT-PCR.

### Xenograft experiments

Male BABL/c nude mice (5 weeks old) were obtained from Charles River Laboratory (Beijing, China) and randomly divided into two groups (*N* = 6 per group). PC3 cells (2 × 10^6^ cells/0.2 mL PBS) stably infected lentivirus-mediated sh-NC or sh-circLRP6 (GeneCopoeia, Rockville, MD, USA) were subcutaneously injected into the right flank of nude mice, respectively, and mice were maintained in Specific Pathogen Free conditions. Tumor size was detected using a caliper every week and tumor volume was calculated with the equation: volume = (length × width^2^)/2. At day 35, tumors were removed and weighted for further molecular analysis with qRT-PCR and western blot, respectively.

### IHC assay

The tumors collected from in vivo tumor formation assay were fixed in formalin and then embedded in paraffin. Paraffin-embedded tumors were deparaffinized in xylene and rehydrated through graded alcohol to water. Then tumor sections were treated with 3% hydrogen peroxide to block endogenous peroxidase activity. After incubation with 5% bovine serum albumin (BSA), the sections were incubated with anti-Ki-67 (ab15580, Abcam), anti-NRBP1 (Cat# H00029959-M01, Abnova) (all 1:100) overnight at 4 °C, secondary antibodies for 1 h at 37 °C and HRP-labeled streptavidin solution for 10 min. Diaminobenzidine (DAB) was used as chromogen.

### Statistical analysis

Data from three repeated experiments were presented as mean ± standard deviation. The data were statistically analyzed using Student’s *t* test (two-sided) or analysis of variance followed by Tukey’ s post hoc analysis. *P* < 0.05 was considered statistically significant.

## Results

### CircLRP6 is highly expressed in PCa tissues and cells

**Fig. 1:**
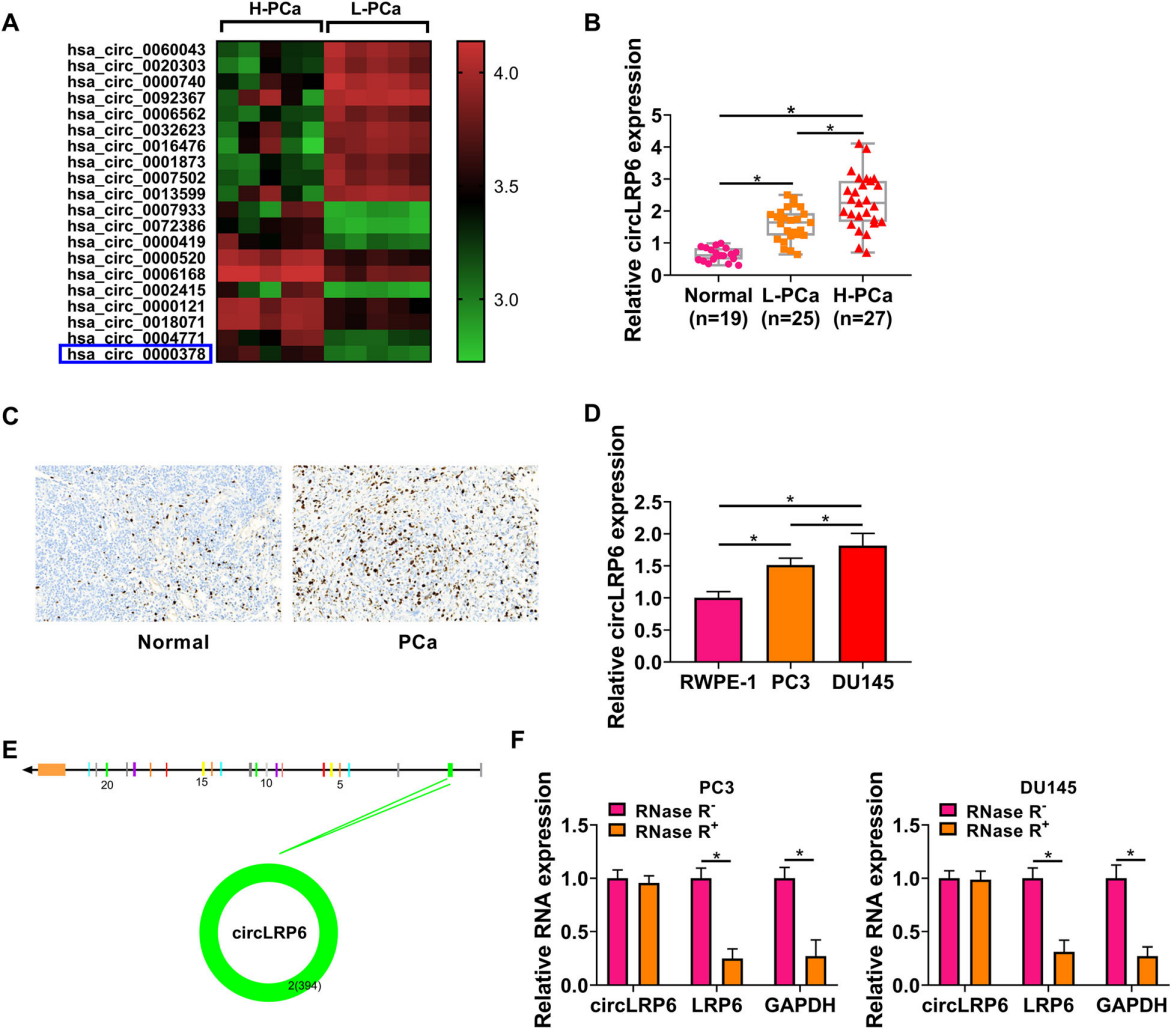
CircLRP6 is highly expressed in PCa tissues and cells. **A** Heat maps showing the top differentially expressed circRNAs. **B** The expression of circLRP6 in normal tissues, low-grade PCa, and high-grade PCa tissues was detected using qRT-PCR. **C** CircLRP6 in PCa tissues or normal tissues was detected by ISH. **D** The expression of circLRP6 in PCa cells (PC3 and DU145) and normal RWPE-1 cells was determined with qRT-PCR. **E** The exonic information of circLRP6 was illustrated and the length of circLRP6 was 394 bp. **F** qRT-PCR analysis of circLRP6 expression in PC3 and DU145 cells treated with or without RNase R. *P < 0.05

According to the public database (GSE113153), multiple circRNAs were found to be differentially expressed in the high-grade and low-grade PCa tissues, among them, circLRP6 expression was found to be significantly higher than other circRNAs (Fig. 1A). Moreover, the expression of circLRP6 was detected in clinical tissues. As expected, circLRP6 was highly expressed in PCa tissues, especially in specimens of patients with high-grade PCa, relative to the normal tissues (Fig. 1B). Moreover, ISH analysis suggested that circLRP6 in PCa tissues was elevated compared with normal tissues (Fig. 1C). In parallel, the expression of circLRP6 was apparently elevated in PCa cells (PC3 and DU145) compared with the RWPE-1 cells (Fig. 1D). CircLRP6 (ID: has_circ_0000378) is derived from exons 2 of the LRP6 gene, whose spliced mature sequence length is 394 bp (Fig. 1E). Next, we investigated the stability of circLRP6 in PCa cells (PC3 and DU145). PC3 and DU145 cells were treated with or without RNase R, which has a 3′-5′ exoribonuclease activity, then it was found that circLRP6 was markedly resistant to RNase R degradation relative to the linear LRP6 mRNA (Fig. 1F), demonstrating that circLRP6 was more stable than LRP6.

### Knockdown of circLRP6 suppresses PCa cell growth and metastasis

**Fig. 2:**
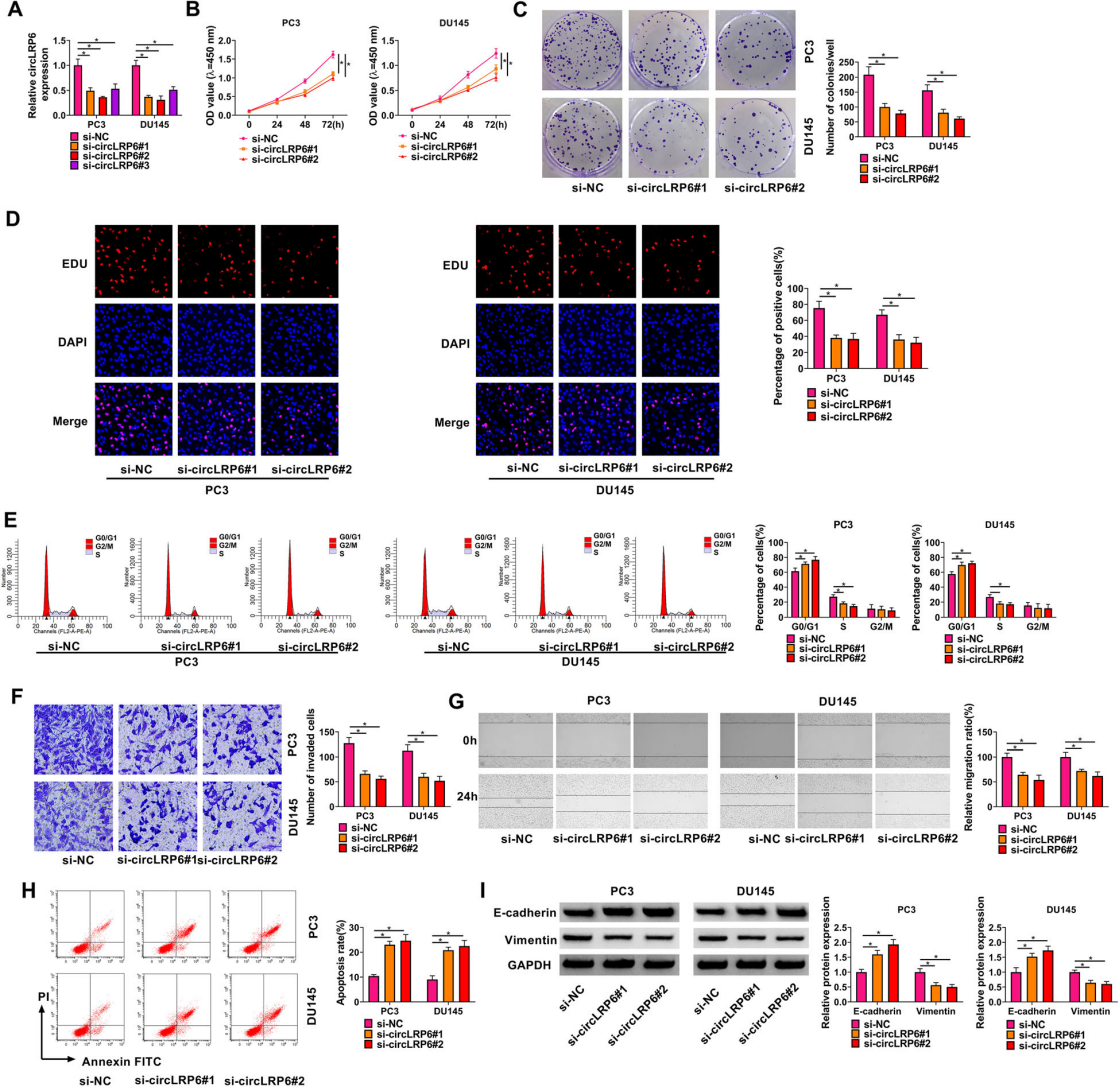
Knockdown of circLRP6 suppresses PCa cell growth and metastasis. **A** Analysis of the interference efficiency of si-circLRP6 (si-circLRP6#1, si-circLRP6#2, or si-circLRP6#3) in PC3 and DU145 cells using qRT-PCR. **B**–**I** PC3 and DU145 cells were transfected with si-circLRP6#1, si-circLRP6#2, or si-NC. **B**–**D** Cell proliferation was determined by CCK-8 assay (**B**), colony formation assay (**C**), and EDU incorporation assay (**D**). **E** Flow cytometry for cell cycle. **F** Transwell assay for cell invasion. **G** Wound healing assay for cell migration. **H** Flow cytometry for cell apoptosis. **I** Western blot analysis of the expression of E-cadherin and Vimentin. *P < 0.05

To determine the biological function of circLRP6 in PCa cells in vitro, short interference siRNAs against circLRP6 (si-circLRP6#1, si-circLRP6#2, si-circLRP6#3) were designed and synthesized to knock down circLRP6 in PC3 and DU145 cells, and the knockdown efficiency was confirmed by qRT-PCR (Fig. 2A).The si-circLRP6#2 and si-circLRP6#3 were selected for subsequent functional analysis. After circLRP6 silencing, the proliferation (Fig. 2B), colony-formation abilities (Fig. 2C), and DNA synthesis activities (Fig. 2D) of PC3 and DU145 cells were markedly decreased, evidenced by the CCK-8, colony formation, and EDU incorporation assays. The results of flow cytometry suggested that silencing of circLRP6 led to an arrest of PC3 and DU145 cells at G0/G1 phase, accompanying by the decreased percentage of cells in S phase (Fig. 2E). In addition, transwell and wound healing assays showed that circLRP6 knockdown suppressed the invasion (Fig. 2F) and migration (Fig. 2G) activities of PC3 and DU145 cells. Besides that, the apoptosis of PC3 and DU145 cells was found to be significantly increased by the knockdown of circLRP6 (Fig. 2H). Moreover, the effects of circLRP6 on EMT, one of major mechanisms for cancer metastasis, were assessed. Western blot analysis showed that E-cadherin was increased while Vimentin was decreased in circLRP6-downregulated PC3 and DU145 cells (Fig. 2I). Taken together, knockdown of circLRP6 suppressed PCa cell growth and metastasis in vitro.

### MiR-330-5p is a target of circLRP6 in PCa cells

**Fig. 3:**
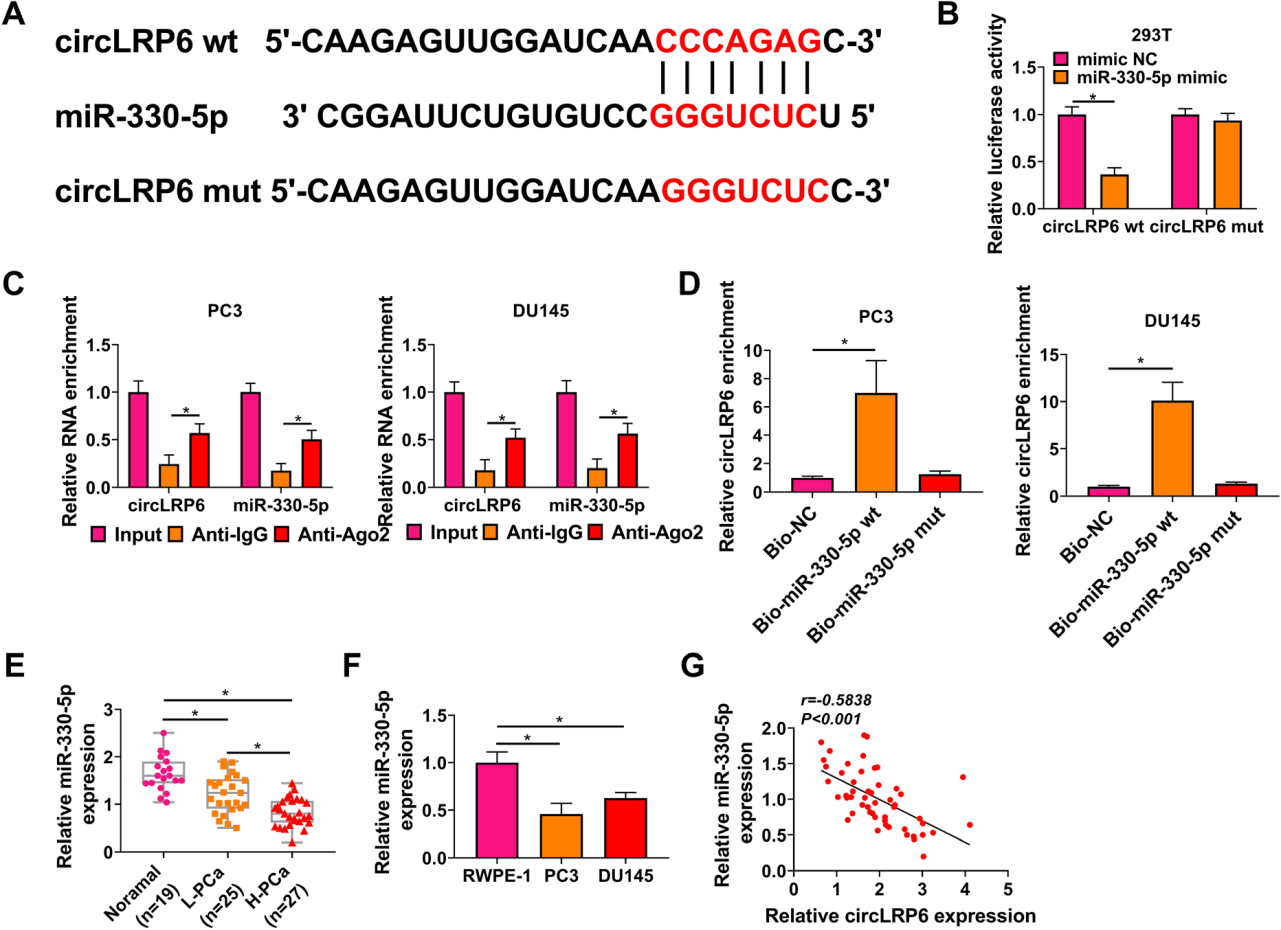
MiR-330-5p is a target of circLRP6 in PCa cells. **A** The putative binding site between circLRP6 and miR-330-5p. **B** Dual-luciferase reporter assay for the relative luciferase activities of the wild and mutated circLRP6 reporter after miR-330-5p upregulation in 293T cells. **C** RIP assay for the examination of circLRP6 and miR-330-5p enrichment in immunoprecipitation complexes in PC3 and DU145 cells. **D** Biotin-labeled pull-down assay indicating the interaction between circLRP6 and miR-330-5p in PC3 and DU145 cells. **E** The expression of miR-330-5p in normal tissues, low-grade PCa and high-grade PCa tissues was detected using qRT-PCR. **F** The expression of miR-330-5p in PCa cells (PC3 and DU145) and normal RWPE-1 cells was determined with qRT-PCR. **G** MiR-330-5p expression was negatively correlated with circLRP6 in PCa tissues. *P < 0.05

The potential targeted miRNAs interacted by circLRP6 were then investigated. Bioinformatics analysis (Circinteractome database) revealed that 12 miRNAs were predicted to contain a putative binding site in circLRP6. Through the search of previous studies and results of qRT-PCR, miR-1247, miR-153, miR-198, miR-326, miR-515-5p, miR-543, and miR-330-5p were revealed to be associated with PCa, among them, the expression levels of miR-198, miR-326, miR-515-5p, miR-543, and miR-330-5p were decreased in PCa, importantly, miR-330-5p expression was significantly affected by circLRP6 knockdown (Fig. S2A-C). Thus, miR-330-5p was selected for subsequent analysis. The putative binding site between circLRP6 and miR-330-5p was shown in Fig. 3A. Then, dual-luciferase reporter assay was carried out. The results suggested that miR-330-5p mimic significantly reduced the luciferase activity of wild-type circLRP6 reporter but not the mutated one in 293T cells (Fig. 3B). Further RIP assay exhibited that the Ago2 antibody could pull down both endogenous circLRP6 and miR-330-5p in PC3 and DU145 cells (Fig. 3C). Moreover, pull-down assay implied that circLRP6 was significantly enriched by Bio-miR-330-5p group compared with Bio-NC group (Fig. 3D). All these results confirmed the direct interaction between circLRP6 and miR-330-5p. After that, the expression profile of miR-330-5p was investigated. As shown in Fig. 3E, miR-330-5p was found to be decreased in PCa tissues, especially in high-grade PCa tissues. Similarly, its expression was also decreased in PCa cells compared with the normal RWPE-1 cells (Fig. 3F). Moreover, a negative correlation between miR-330-5p and circLRP6 expression was observed in PCa tissues (Fig. 3G). In all, these results verified that miR-330-5p was an inhibitory target of circLRP6 in PCa cells.

### Knockdown of circLRP6 suppresses PCa cell growth and metastasis via targeting miR-330-5p

**Fig. 4:**
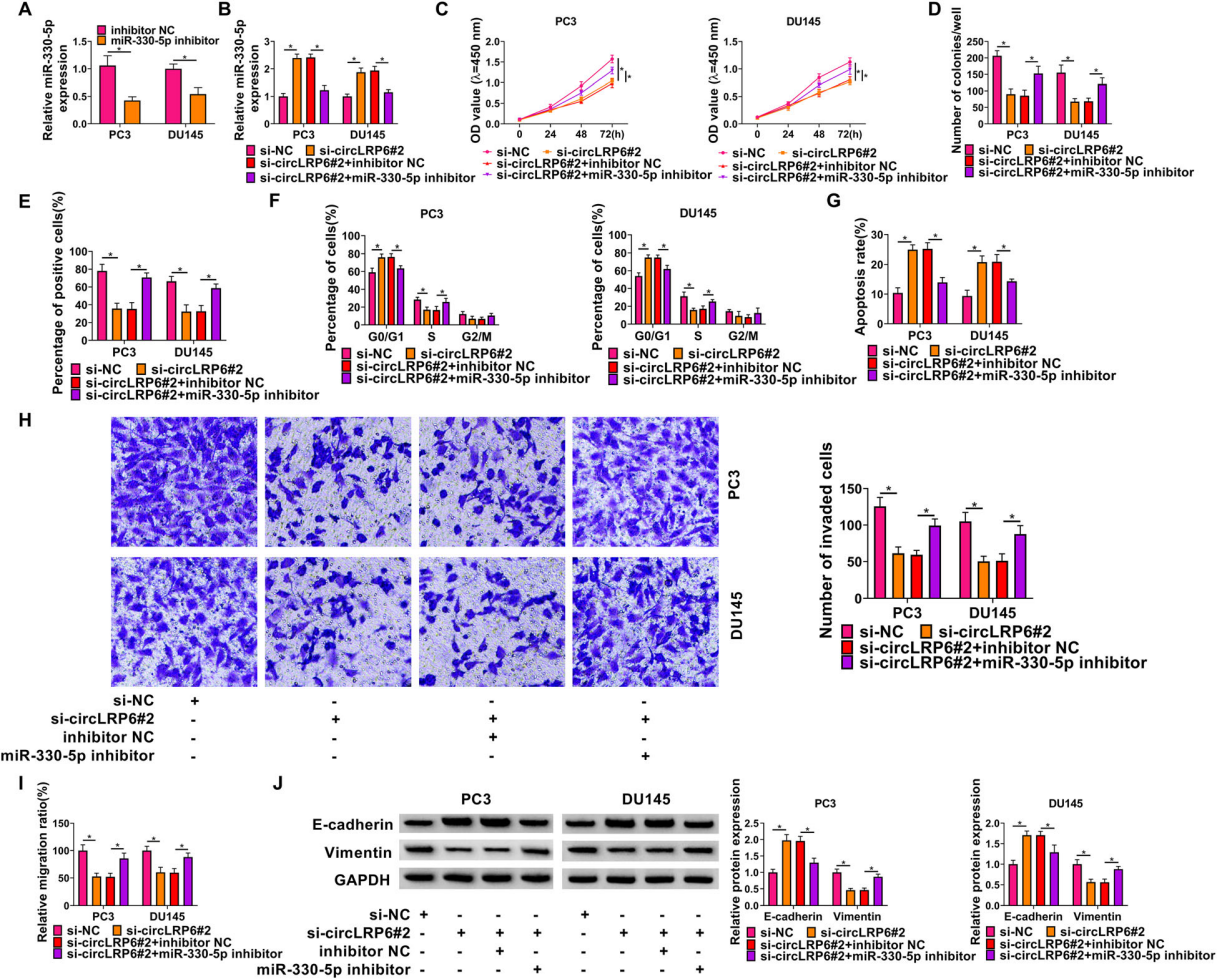
Knockdown of circLRP6 suppresses PCa cell growth and metastasis via targeting miR-330-5p. **A** Analysis of the interference efficiency of miR-330-5p inhibitor or inhibitor NC using qRT-PCR. **B**–**J** PC3 and DU145 cells were transfected with si-NC, si-circLRP6#2, si-circLRP6#2 + inhibitor NC, or si-circLRP6#2 + miR-330-5p inhibitor. **B** qRT-PCR of miR-330-5p expression in cells. **C**–**E** Cell proliferation were determined by CCK-8 assay (**C**), colony formation assay (**D**), and EDU incorporation assay (**E**). **F**, **G** Flow cytometry for cell cycle and apoptosis. **H** Transwell assay for cell invasion. **I** Wound healing assay for cell migration. **J** Western blot analysis of the expression of E-cadherin and Vimentin. *P < 0.05

Next, we examined whether miR-330-5p mediated the action of circLRP6 on PCa cells. qRT-PCR analysis suggested that the introduction of miR-330-5p inhibitor remarkably reduced the expression level of miR-330-5p in PC3 and DU145 cells compared with the negative control (Fig. 4A). Then miR-330-5p inhibitors were transfected into circLRP6-decreased PC3 and DU145 cells, as expected, miR-330-5p inhibitors reversed circLRP6 knockdown-induced elevation of miR-330-5p in cells (Fig. 4B). Thereafter, rescue assay was performed. It was proved that miR-330-5p inhibition attenuated circLRP6 knockdown-evoked suppression of cell proliferation (Fig. 4C), colony-formation abilities (Fig. 4D), and DNA synthesis activities (Fig. 4E) in PC3 and DU145 cells. The arrest of cell cycle (Fig. 4F) and reduction of cell apoptosis (Fig. 4G) induced by circLRP6 knockdown were partially abolished by the inhibition of miR-330-5p. Furthermore, transfection of miR-330-5p inhibitors reversed circLRP6 knockdown-mediated cell metastasis inhibition, reflected by the increase of the invaded and migrated PC3 and DU145 cells (Fig. 4H, I), as well as the progression of EMT in PC3 and DU145 cells (Fig. 4J). Additionally, we also determined the effects of circLRP6/miR-330-5p axis on normal RWPE-1 cells. As shown in Fig. S1A, miR-330-5p inhibitor also reduced circLRP6 knockdown-induced increase of miR-330-5p expression in RWPE-1 cells. Then it was found that the circLRP6/miR-330-5p axis had no effects on RWPE-1 cells (Fig. S1B–D). Altogether, circLRP6 affected PCa cell growth and metastasis via regulating miR-330-5p.

### MiR-330-5p directly targets NRBP1 in PCa cells

**Fig. 5:**
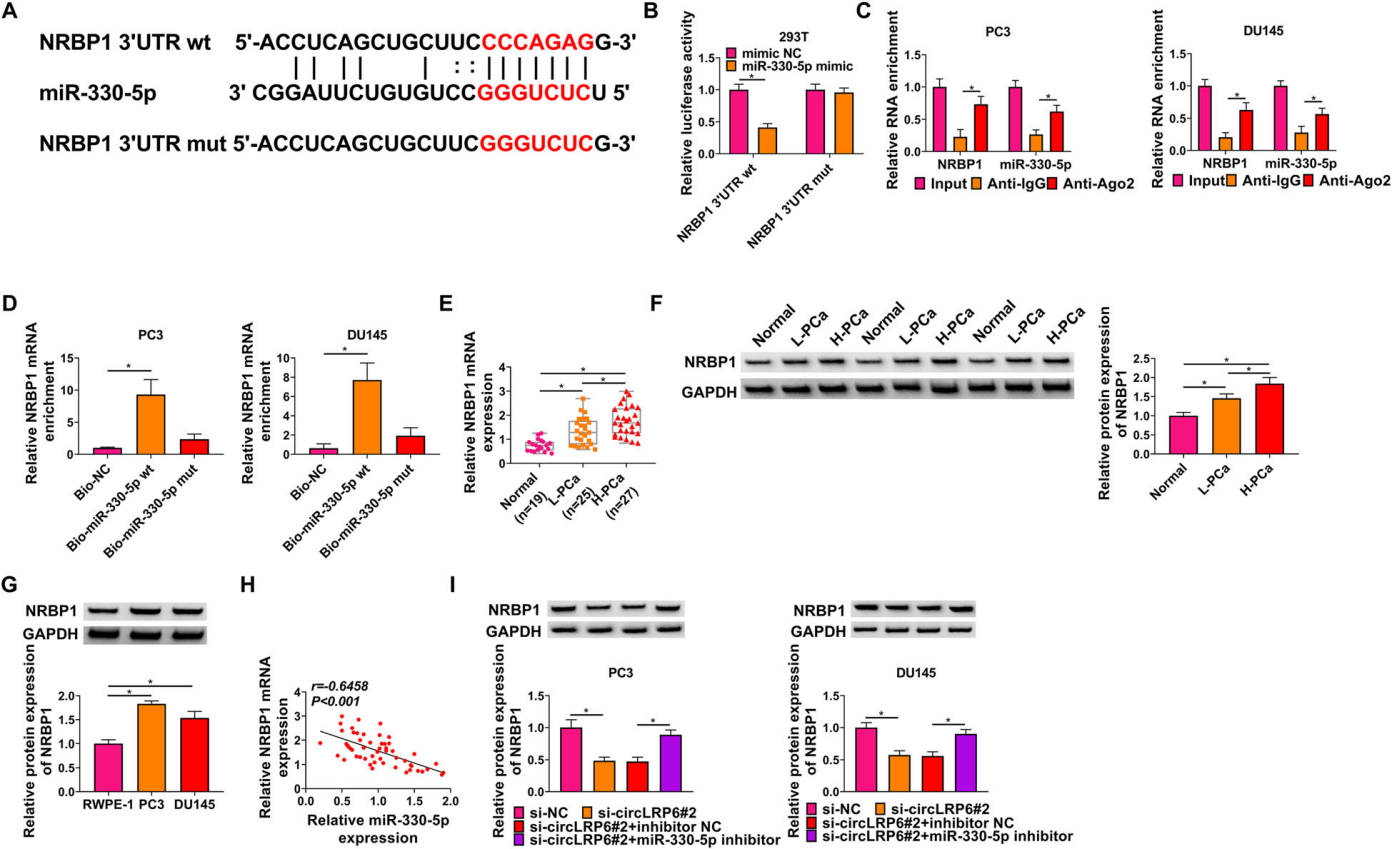
MiR-330-5p directly targets NRBP1 in PCa cells. **A** The sequence of miR-330-5p binding sites within NRBP1. **B** Dual-luciferase reporter assay for the detection of the relative luciferase activities of wild and mutated NRBP1 reporter after miR-330-5p upregulation in 293T cells. **C** RIP assay for the examination of NRBP1 and miR-330-5p RNA enrichment in immunoprecipitation complexes in PC3 and DU145 cells. **D** Biotin-labeled pull-down assay indicating the interaction between NRBP1 and miR-330-5p in PC3 and DU145 cells. **E**, **F** The expression of NRBP1 in normal tissues, low-grade PCa, and high-grade PCa tissues was detected using qRT-PCR and western blot assay. **G** The expression of NRBP1 in PCa cells (PC3 and DU145) and normal RWPE-1 cells was determined with western blot. **H** NRBP1 expression was negatively correlated with miR-330-5p in PCa tissues. **I** Western blot analysis of NRBP1 expression in PC3 and DU145 cells transfected with si-NC, si-circLRP6#2, si-circLRP6#2 + inhibitor NC, or si-circLRP6#2 + miR-330-5p inhibitor. *P < 0.05

Based on the results of bioinformatics analysis, numerous genes were predicted to have the binding site of miR-330-5p, among which, IGF1R, MYO6, SMAD3, CCND1, and NRBP1 were selected due to their high expression in PCa according the previous researches. Thereafter, western blot analysis showed that NRBP1 expression was overtly reduced by miR-330-5p up-regulation (Fig. S2C, D). The putative binding site of NRBP1 and miR-330-5p was exhibited in Fig. 5A. The results of dual-luciferase reporter assay showed that miR-330-5p mimics reduced the luciferase activity in the wild-type rather than mutated type of NRBP1 in 293T cells (Fig. 5B). RIP assay further exhibited that the levels of miR-330-5p and NRBP1 were visibly enriched in anti-Ago2 group compared with that in anti-IgG group in PC3 and DU145 cells (Fig. 5C). Pull-down assay indicated that the enrichment level of NRBP1 in the Bio-miR-330-5p group was significantly higher than that in the Bio-NC group (Fig. 5D), further confirming the binding between miR-330-5p and NRBP1. Thereafter, NRBP1 expression was found to be highly expressed in high-grade PCa tissues in comparison to the low-grade and normal PCa tissues (Fig. 5E, F), as well as in PCa cells compared with the normal RWPE-1 cells (Fig. 5G). Moreover, its expression was negatively correlated with miR-330-5p in PCa tissues (Fig. 5H). Further western blot analysis suggested that circLRP6 knockdown reduced the level of NRBP1 in PC3 and DU145 cells, which was rescued by the inhibition of miR-330-5p (Fig. 5I). Thus, we confirmed that miR-330-5p targeted NRBP1 and circLRP6 could regulate NRBP1 expression by sponging miR-330-5p.

### MiR-330-5p restrains PCa cell growth and metastasis via targeting NRBP1

**Fig. 6:**
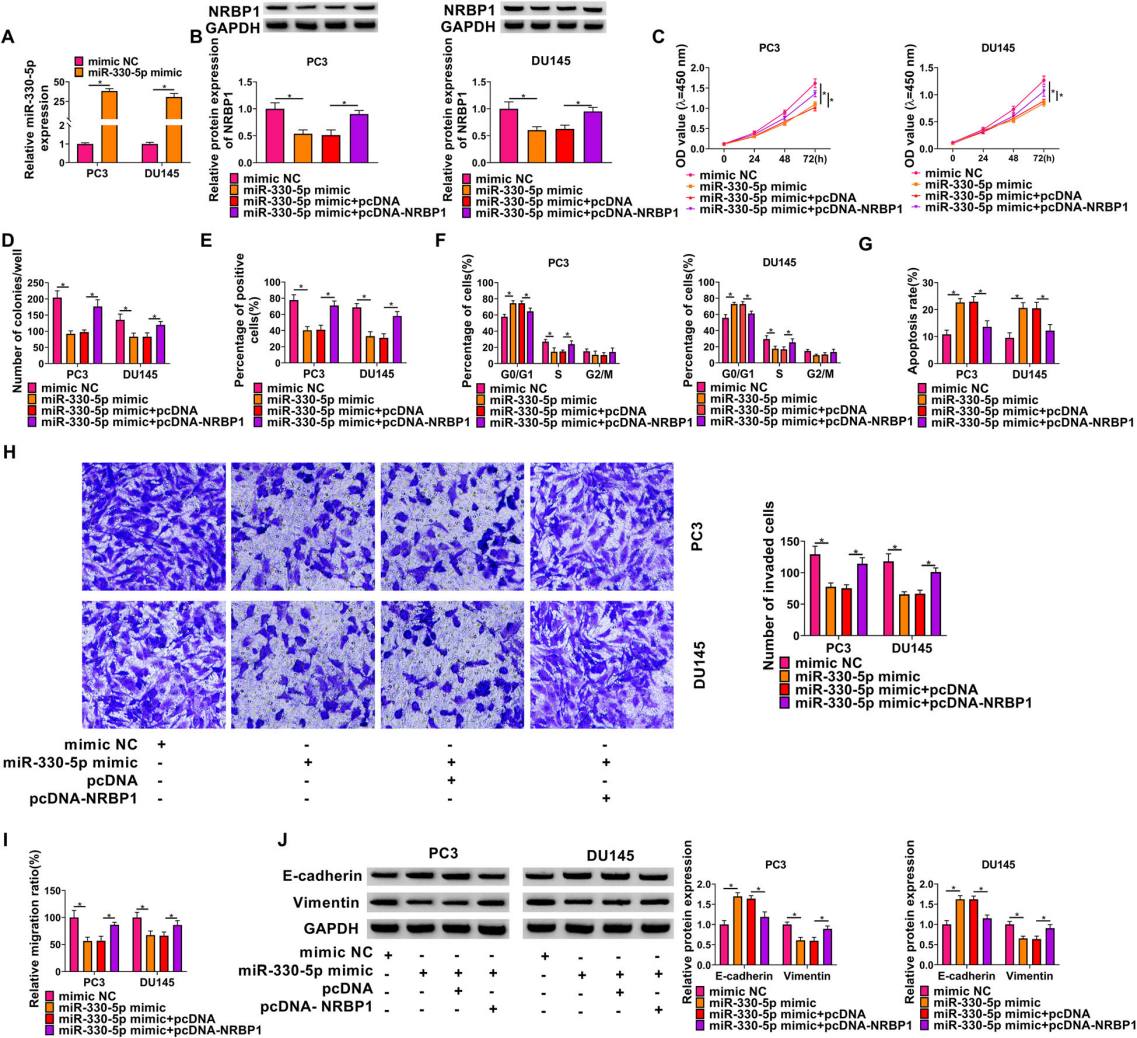
MiR-330-5p restrains PCa cell growth and metastasis via targeting NRBP1. **A** qRT-PCR analysis of miR-330-5p expression in PC3 and DU145 cells transfected with miR-330-5p mimic or mimic NC. **B**–**J** PC3 and DU145 cells were transfected with mimic NC, miR-330-5p mimic, miR-330-5p mimic + pcDNA, or miR-330-5p mimic + pcDNA-NRBP1. **B** Western blot analysis of NRBP1 expression in cells. **C**–**E** Cell proliferation were determined by CCK-8 assay (**C**), colony formation assay (**D**), and EDU incorporation assay (**E**). **F**, **G** Flow cytometry for cell cycle and apoptosis. **H** Transwell assay for cell invasion. **I** Wound healing assay for cell migration. **J** Western blot analysis of the expression of E-cadherin and Vimentin. *P < 0.05

We then investigated whether miR-330-5p mediated PCa cell growth and metastasis via NRBP1. qRT-PCR analysis showed that miR-330-5p mimics significantly elevated miR-330-5p in PC3 and DU145 cells (Fig. 6A). Then PC3 and DU145 cells were co-transfected with miR-330-5p mimic and NRBP1 overexpressing pcDNA to perform rescue assay. Western blot analysis indicated miR-330-5p mimic reduced the expression level of NRBP1 in PC3 and DU145 cells, which was rescued by the transfection of pcDNA-NRBP1 (Fig. 6B). CCK-8, colony formation, and EDU incorporation assays were used to detect cell proliferation. Results showed that miR-330-5p mimic led to the increase of the proliferation rate (Fig. 6C), colony-formation abilities (Fig. 6D), and DNA synthesis activities (Fig. 6E) in PC3 and DU145 cells, which were reversed by the overexpression of NRBP1 (Fig. 6C–E). Flow cytometry revealed that NRBP1 overexpression attenuated miR-330-5p mimic-mediated cell cycle arrest and cell apoptosis suppression in PC3 and DU145 cells (Fig. 6F, G). Furthermore, we also demonstrated that miR-330-5p mimic suppressed PC3 and DU145 cell to invade, migrate and EMT, while this condition was counteracted by NRBP1 overexpression (Fig. 6H–J). Collectively, miR-330-5p/NRBP1 axis was engaged in PCa cell growth and metastasis.

### CircLRP6 knockdown impedes PCa tumor growth and EMT in vivo

**Fig. 7:**
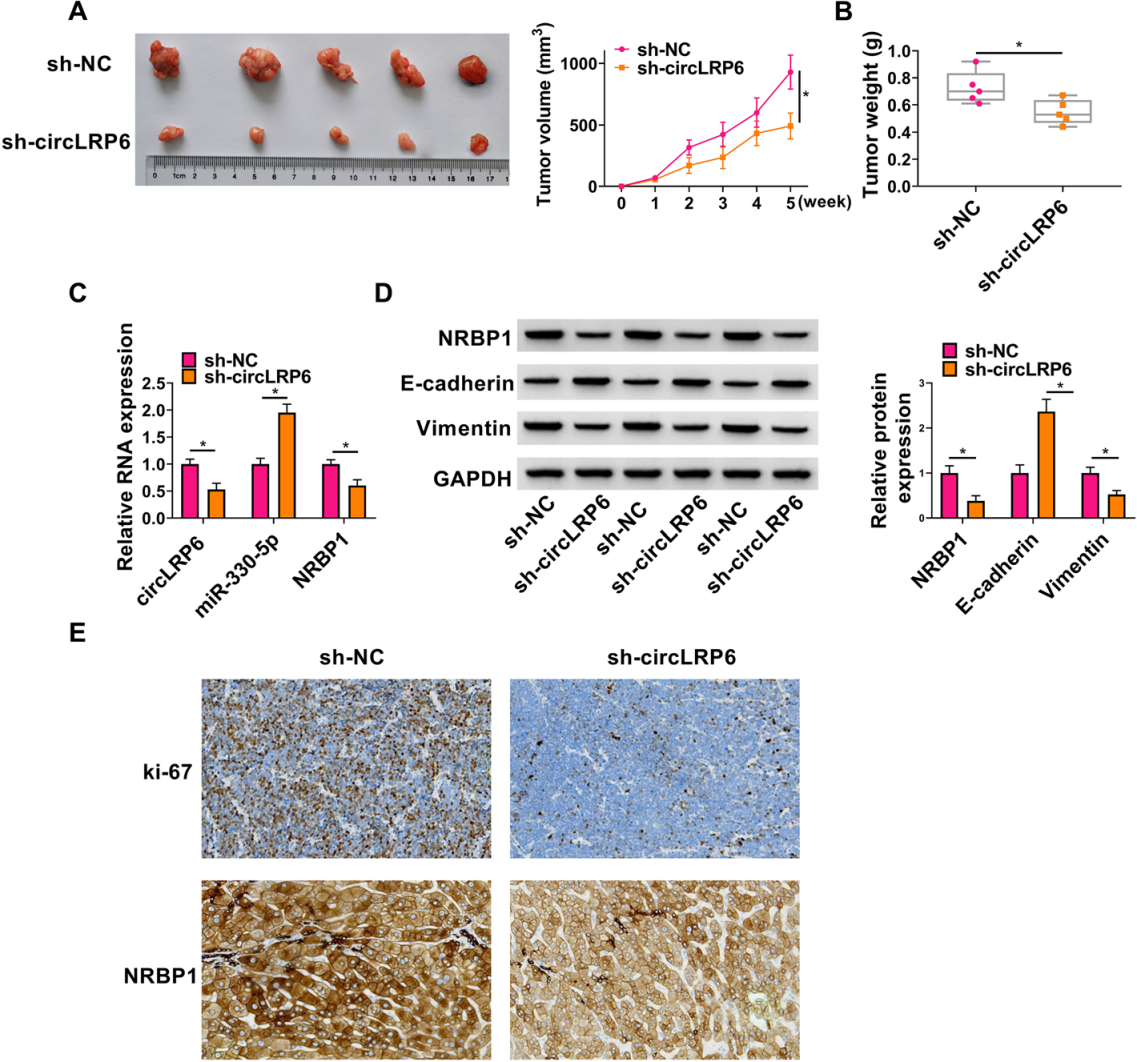
CircLRP6 knockdown impedes PCa tumor growth and EMT in vivo. A subcutaneous tumor model was established. **A** Image of tumors (left panel) and in vivo growth curve (right panel). **B** Tumor weight of each group was analyzed at day 35. **C** qRT-PCR analysis of circLRP6, NRBP1, and miR-330-5p expression in tumors of mice from each group. **D** Western blot analysis of NRBP1, E-cadherin, and Vimentin protein levels in the tumor of mice from each group. **E** Representative images of IHC staining of NRBP and Ki-67 in tumors. *P < 0.05

Subsequently, the biological function of circLRP6 in vivo was explored using the PC3 tumor model with BABL/c nude mice. Consistent with the results in vitro, circLRP6 silencing prominently decreased tumor volume and weight compared with those in the sh-NC group (Fig. 7A, B). Molecular analysis showed that the expression of circLRP6 and NRBP1 was lower, while miR-330-5p expression was higher in tumors isolated from sh-circLRP6 groups (Fig. 7C, D). The results of western blot analysis suggested that circLRP6 knockdown led to an increase of E-cadherin protein level and a decrease of Vimentin protein level in tumors of mice (Fig. 7D). Furthermore, IHC assay confirmed that the expression of Ki-67 and NRBP1 was downregulated in tumors of sh-circLRP6 group compared with sh-NC group (Fig. 7E). These results suggested that circLRP6 knockdown suppressed PCa tumor growth and EMT in vivo.

## Discussion

Recently, aberrant expression of circRNAs has been shown in PCa, and circRNAs have been revealed to play significant roles in PCa progression [19, 20]. The functions and regulation of circRNAs have attracted great research interest for discovering novel targets in the diagnosis and treatment of PCa. In the present study, through the use of circular RNA microarrays, circLRP6 was found to be highly expressed in PCa tissues. Then, the uncharacterized circLRP6 in PCa tumorigenesis was investigated. As expected, circLRP6 expression also was higher in clinical PCa tissues, especially in high-graded PCa tissues. In vitro functional studies indicated that knockdown of circLRP6 could suppress PCa cell proliferation, invasion, migration, but induce apoptosis. E-cadherin is a transmembrane glycoprotein, involved in cell-cell adhesion and EMT. Vimentin is highly expressed in mesenchymal cells and positively correlated with increased metastasis [21]. In this study, the results of western blot analysis suggested that circLRP6 silencing reduced Vimentin expression and increased E-cadherin expression. Therefore, we demonstrated that knockdown of circLRP6 suppressed PCa cell growth and metastasis in vitro. Importantly, consistent with the results in vitro, results of tumor formation assay confirmed that circLRP6 silencing prominently decreased tumor growth and EMT in vivo. All these results indicated a tumor-promoter role of circLRP6 in PCa.

Mounting evidence suggests that circRNAs can serve as endogenous sponges of miRNAs to protect the target mRNAs from repression [22, 23]. Wang et al. revealed that circITCH worked as a sponge in PCa, regulated the expression of miR-17-5p to increase HOXB13 expression level and repressed malignant phenotype of PCa [24]. Yang et al. showed circAMOTL1L increased Pcdha expression by sponging miR-193a-5p and suppressed migration and invasion of PCa cells through leading to EMT inhibition [25]. In the present study, we also confirmed that circLRP6 acted as a sponge for miR-330-5p to upregulate NRBP1 expression in PCa in vitro and in vivo. However, whether circLRP6 exerted its action via miR-330-5p/NRBP1 axis remains unclear.

MiRNAs are one kind of noncoding RNAs composed of 21–23 nucleotides, which have been reported to involve in the progression of many types of cancers though modulating cellular biological processes such as cell proliferation, migration, and apoptosis [26, 27]. Current treatments for PCa mainly include surgery, chemotherapy, radiotherapy, androgen deprivation therapy, and castration [28], Egidi et al. showed that miRNAs, including miR-21 and miR-141, could be implicated in postsurgical inflammatory processes and did not impacted by radical prostatectomy [29]. Besides that, miRNAs, in serum or urine exfoliated cell, were demonstrated to be reliable invasive biomarkers for the diagnosis and prognosis of PCa [28, 30]. Moreover, they were also discovered to be stable in post-digital rectal examination (DRE) urine sediments and might be used as reference genes for the assessment of suspected PCa [31]. In the meanwhile, numerous miRNAs are revealed to participate in the tumorigenesis of PCa [32]. Therefore, abnormalities of miRNAs expression may be promising biomarkers for the prediction, diagnosis, and treatment in PCa.

MiR-330-5p has been proven to be connected with diverse cancers. However, the function of miR-330-5p is complicated because it can be a tumor suppressor or a carcinogen in the context of different cancers. MiR-330-5p is identified as tumor suppressor in osteosarcoma by suppressing cell growth and invasion through the inhibition of the proto-oncogene survivin [33]. In glioblastoma, miR-330-5p suppressed ITGA5 expression to impede cell proliferation and invasion [34]. Besides that, miR-330-5p repressed cell invasive phenotype through downregulating MMP1 expression in esophageal adenocarcinoma [35]. Additionally, miR-330-5p was demonstrated to function as onco-miR in cervical cancer by enhancing cancer cell invasion and metastasis [36]. Moreover, miR-330-5p was confirmed to suppress SPRY2 to promote proliferation via MAPK/ERK signaling in hepatocellular carcinoma [37]. In PCa, miR-330-5p was demonstrated to restrain PCa growth and metastasis through the LEF1-AS1/miR-330-5p/LEF1 pathway in vitro and in vivo [38]. All these findings suggested the involvement of miR-330-5p in cancer growth and metastasis.

MiRNA can regulate gene expression via binding to the 3′UTR of the target mRNA at the level of mRNA turnover or translation, thereby exerting their effects in carcinogenesis [27, 39, 40]. NRBP1 is a ubiquitously expressed, highly conserved pseudokinase [41]. NRBP1 has been revealed to exert different functions in different cancers [42, 43]. In PCa, high NRBP1 expression could be of prognostic value in PCa patients, and enhanced cancer cell proliferation [44]. Besides, Yan et al. showed NRBP1 was involved in miR-519d-induced inhibition of PCa cell growth and invasion [45]. Thus, NRBP1 is considered to be an oncogene in PCa. In the current study, a mutual antagonism between miR-330-5p and circLRP6 on affecting PCa cell growth and metastasis was investigated. Importantly, we also demonstrated that miR-330-5p overexpression suppressed the progression of PCa, which was attenuated by NRBP1 upregulation. Taken together, a functional regulatory network in PCa growth and metastasis was identified.

## Conclusion

In conclusion, our findings suggested that circLRP6 increased NRBP1 expression level by miR-330-5p, subsequent promoted PCa growth and metastasis, suggesting a new direction for PCa treatment.

## Supplementary Information

**Additional file 1: Figure S1.** The effects of circLRP6/miR-330-5p axis on normal RWPE-1 cells. (**A-D**) RWPE-1 cells were transfected with si-NC, si-circLRP6#2, si-circLRP6#2 + inhibitor NC, or si-circLRP6#2 + miR-330-5p inhibitor. (**A**) qRT-PCR of miR-330-5p expression in cells. (**B**) Cell proliferation were determined by colony formation assay. (**C**) Flow cytometry for cell apoptosis. (**D**) Transwell assay for cell invasion. *P<0.05.

**Additional file 2: Figure S2.** The effects of circLRP6 or miR-330-5p on the expression levels of potential target genes. (**A**) qRT-PCR of miR-1247, miR-153, miR-198, miR-326, miR-515-5p, miR-543, and miR-330-5p expression levels in PCa cells (PC3 and DU145) and normal RWPE-1 cells. (**B, C**) qRT-PCR of miR-198, miR-326, miR-515-5p, miR-543, and miR-330-5p expression levels in PC3 and DU145 cells transfected with si-NC or si-circLRP6#2. (**D**) Western blot analysis of the protein levels of IGF1R, MYO6, SMAD3, CCND1, NRBP1 in PC3 and DU145 cells transfected with mimic NC or miR-330-5p mimic. *P<0.05.

## Data Availability

The data sets used and/or analyzed during the current study are available from the corresponding author on reasonable request.
