# Supplementary figures and images for: CircLRP6 contributes to prostate cancer growth and metastasis by binding to miR-330-5p to up-regulate NRBP1
Source: World J Surg Oncol. 2021 Jun 22;19:184. doi: 10.1186/s12957-021-02287-2 (PMC8220703; doi:10.1186/s12957-021-02287-2)

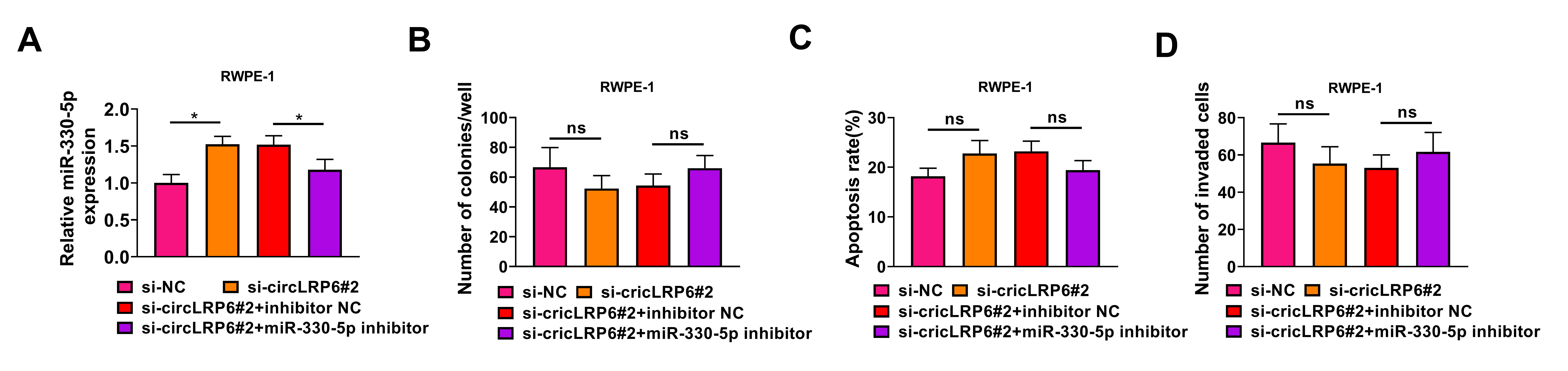

Supplement: Supplementary file 1 — Additional file 1: Figure S1. The effects of circLRP6/miR-330-5p axis on normal RWPE-1 cells. (A-D) RWPE-1 cells were transfected with si-NC, si-circLRP6#2, si-circLRP6#2 + inhibitor NC, or si-circLRP6#2 + miR-330-5p inhibitor. (A) qRT-PCR of miR-330-5p expression in cells. (B) Cell proliferation were determined by colony formation assay. (C) Flow cytometry for cell apoptosis. (D) Transwell assay for cell invasion. *P<0.05. [file 12957_2021_2287_MOESM1_ESM.jpg]

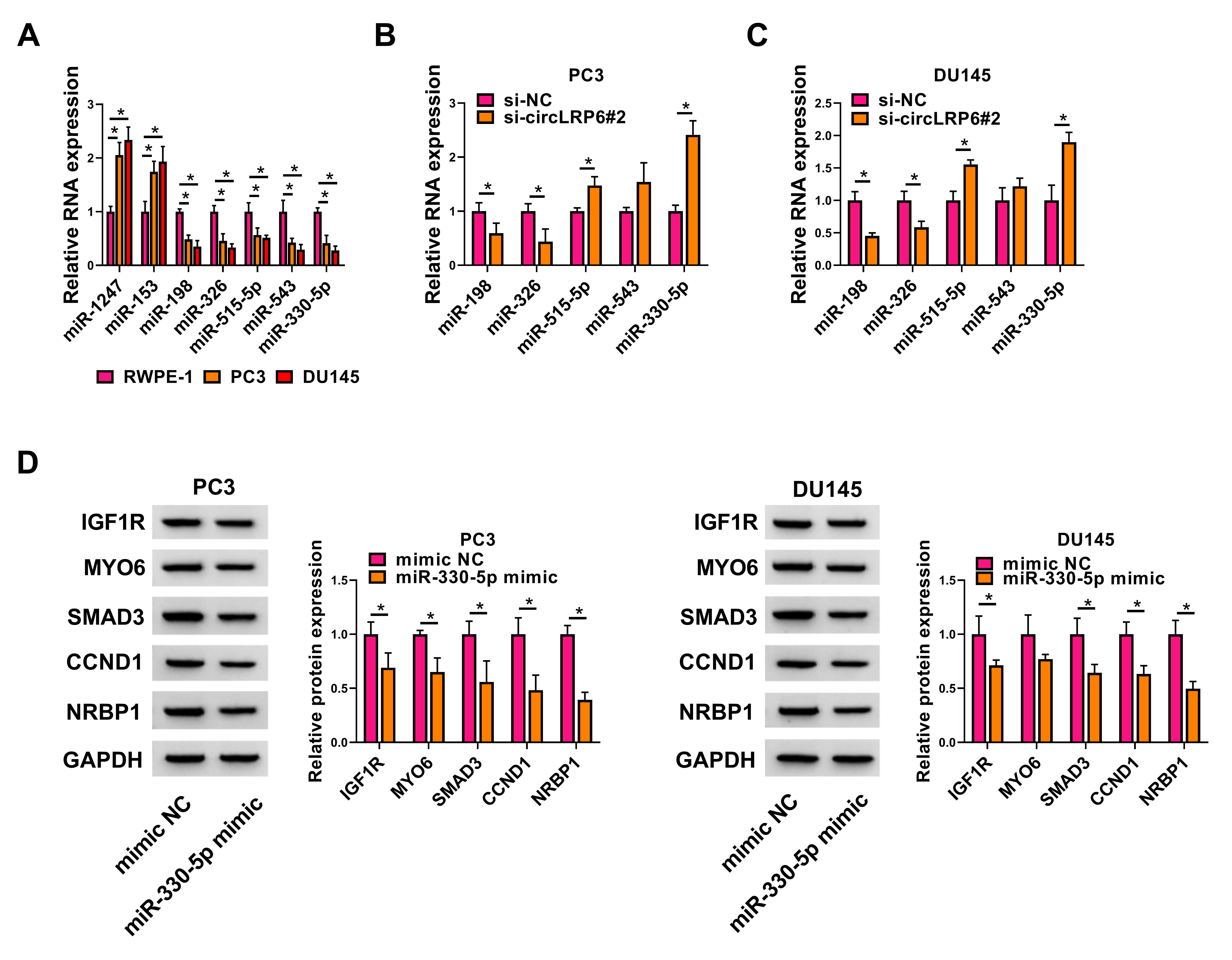

Supplement: Supplementary file 2 — Additional file 2: Figure S2. The effects of circLRP6 or miR-330-5p on the expression levels of potential target genes. (A) qRT-PCR of miR-1247, miR-153, miR-198, miR-326, miR-515-5p, miR-543, and miR-330-5p expression levels in PCa cells (PC3 and DU145) and normal RWPE-1 cells. (B, C) qRT-PCR of miR-198, miR-326, miR-515-5p, miR-543, and miR-330-5p expression levels in PC3 and DU145 cells transfected with si-NC or si-circLRP6#2. (D) Western blot analysis of the protein levels of IGF1R, MYO6, SMAD3, CCND1, NRBP1 in PC3 and DU145 cells transfected with mimic NC or miR-330-5p mimic. *P<0.05. [file 12957_2021_2287_MOESM2_ESM.jpg]
